# Intracluster correlation coefficients in the Greater Mekong Subregion for sample size calculations of cluster randomized malaria trials

**DOI:** 10.1186/s12936-019-3062-x

**Published:** 2019-12-18

**Authors:** Pimnara Peerawaranun, Jordi Landier, Francois H. Nosten, Thuy-Nhien Nguyen, Tran Tinh Hien, Rupam Tripura, Thomas J. Peto, Koukeo Phommasone, Mayfong Mayxay, Nicholas P. J. Day, Arjen Dondorp, Nick White, Lorenz von Seidlein, Mavuto Mukaka

**Affiliations:** 1 Mahidol Oxford Tropical Medicine Research Unit, Faculty of Tropical Medicine, Mahidol University, 60th Anniversary Chalermprakiat Building, 3rd Floor, 420/6 Ratchawithi Rd, Ratchathewi District, Bangkok, 10400 Thailand; 2 Shoklo Malaria Research Unit, Mahidol-Oxford Tropical Medicine Research Unit, Faculty of Tropical Medicine, Mahidol University, Mae Sot, Thailand; 3 Aix-Marseille University, IRD, INSERM, SESSTIM, Marseille, France; 4 Centre for Tropical Medicine and Global Health, Nuffield Department of Medicine, University of Oxford, Oxford, UK; 5 Oxford University Clinical Research Unit, Wellcome Trust Major Oversea Programme, Ho Chi Minh City, Vietnam; 6 Department of Global Health, Amsterdam University Medical Centers, Location AMC, Amsterdam, The Netherlands; 7 Lao-Oxford-Mahosot Hospital-Wellcome Trust Research Unit (LOMWRU), Microbiology Laboratory, Mahosot Hospital, Vientiane, Lao PDR; 8 Amsterdam Institute for Global Health & Development, Amsterdam, Netherlands; 9 Institute of Research and Education Development, University of Health Sciences, Vientiane, Lao PDR

**Keywords:** ICC, Malaria, Prevalence, Incidence, Cluster randomized trial, Sample size, *P. falciparum*, *P. vivax*, Bootstrapping

## Abstract

**Background:**

Sample size calculations for cluster randomized trials are a recognized methodological challenge for malaria research in pre-elimination settings. Positively correlated responses from the participants in the same cluster are a key feature in the estimated sample size required for a cluster randomized trial. The degree of correlation is measured by the intracluster correlation coefficient (ICC) where a higher coefficient suggests a closer correlation hence less heterogeneity within clusters but more heterogeneity between clusters.

**Methods:**

Data on uPCR-detected *Plasmodium falciparum* and *Plasmodium vivax* infections from a recent cluster randomized trial which aimed at interrupting malaria transmission through mass drug administrations were used to calculate the ICCs for prevalence and incidence of *Plasmodium* infections. The trial was conducted in four countries in the Greater Mekong Subregion, Laos, Myanmar, Vietnam and Cambodia. Exact and simulation approaches were used to estimate ICC values for both the prevalence and the incidence of parasitaemia. In addition, the latent variable approach to estimate ICCs for the prevalence was utilized.

**Results:**

The ICCs for prevalence ranged between 0.001 and 0.082 for all countries. The ICC from the combined 16 villages in the Greater Mekong Subregion were 0.26 and 0.21 for *P*. *falciparum* and *P. vivax* respectively. The ICCs for incidence of parasitaemia ranged between 0.002 and 0.075 for Myanmar, Cambodia and Vietnam. There were very high ICCs for incidence in the range of 0.701 to 0.806 in Laos during follow-up.

**Conclusion:**

ICC estimates can help researchers when designing malaria cluster randomized trials. A high variability in ICCs and hence sample size requirements between study sites was observed. Realistic sample size estimates for cluster randomized malaria trials in the Greater Mekong Subregion have to assume high between cluster heterogeneity and ICCs. This work focused on uPCR-detected infections; there remains a need to develop more ICC references for trials designed around prevalence and incidence of clinical outcomes. Adequately powered trials are critical to estimate the benefit of interventions to malaria in a reliable and reproducible fashion.

*Trial registration*: ClinicalTrials.govNCT01872702. Registered 7 June 2013. Retrospectively registered. https://clinicaltrials.gov/ct2/show/NCT01872702

## Background

In malaria elimination, many intervention strategies must be evaluated at a cluster level to estimate the impact on transmission. In vector borne diseases like malaria the unit of randomization tends to be geographically defined (e.g. household or village), but can also be sub-populations such as children attending a school [1]. In general, trials that use individual randomization are statistically more efficient than cluster randomized trials (CRTs) because the responses of individuals belonging to the same cluster tend to be more closely correlated than responses of individuals belonging to different clusters [1–3]. The degree of correlation is quantified by the intracluster correlation coefficient (ICC). The design and analysis of CRTs must account for the ICC as statistical methods designed for individually randomized trials fail to account for these correlations. Sample sizes required for CRTs must be inflated to obtain the appropriate statistical power [1, 4, 5]. Formulas for calculating sample sizes for CRTs have been published [1, 2, 4–8] and are integrated in statistical software packages such as Stata, PASS and R. The basic formulae for sample size calculation in CRTs is the sample size of an individually randomized trial multiplied by an inflation factor called design effect (DEff), also known as variance inflation factor (VIF) to account for clustering in the CRT design [1]. The elements of the inflation factor are the ICC ($\rho$), and the cluster size, $m$, giving a ${\text{DEff}}\, = \,{\text{VIF}}\, = \,1 + \left( {m - 1} \right)\rho$. Thus, ICC is a key element in sample size calculations for cluster randomized trials.

The basic definition of ICC is $\rho = \frac{{\sigma_{b}^{2} }}{{\sigma_{b}^{2} + \sigma_{w}^{2} }}$, where $\sigma_{b}^{2}$ is the variance of the outcome between clusters and $\sigma_{w}^{2}$ is the variance of the outcome within clusters. The sum of $\sigma_{b}^{2}$ and $\sigma_{w}^{2}$ gives the total variance for a cluster randomized trial outcome. Thus, the ICC is the proportion of the total variance of an outcome that can be explained by the between cluster variation in the outcome. The ICC can be estimated from earlier studies of similar nature as the planned trial. It can be challenging to find relevant ICC values for sample size calculations [6, 9]. Unfortunately, researchers frequently omit the estimation of ICC as a secondary outcome in protocols and reports. Furthermore, it can be challenging to estimate the ICC accurately in multilevel models which are increasingly used for the analysis of cluster randomized trials. The main challenge in estimating ICCs for the discrete models such as Poisson regression is that the ICC are not constant across the data rather they depend on the fixed part of the model [8, 10, 11].

The objective of this study was to utilize the data from recent mass drug administrations in the GMS to estimate overall and country specific ICC values for the prevalence and incidence outcomes of *Plasmodium falciparum* and *Plasmodium vivax* infections [12] to aid in the design of future cluster randomized malaria trials.

## Methods

The ICCs have been estimated from the data that was generated in the Targeted Malaria Elimination study (TME) with mass drug administrations (MDA) on falciparum malaria in South-East Asia [12]. Following vector control activities, community-based case management and intensive community engagement, restricted randomization was conducted within village pairs to select 8 villages to receive early MDA and 8 villages as controls. After 12 months the control villages received deferred MDA. The MDA comprised 3 monthly rounds of 3 daily doses of dihydroartemisinin–piperaquine and, except in Cambodia, a single low dose of primaquine. Cross-sectional surveys of the entire population of each village at quarterly intervals using ultrasensitive quantitative PCR (uPCR) were used to detect *Plasmodium* infections. The overall aim of the study was to assess the duration of effectiveness of MDA on falciparum parasitaemia incidence and prevalence in 16 remote village populations, 4 villages each in Myanmar, Vietnam, Cambodia and Laos. The sample size, 4 village clusters per country, was chosen mainly for operational and practical reasons. The detailed methods of the TME study have been published [12–16].

### Definitions of outcomes

Defining a new *P. vivax* infection from longitudinally collected data is more complicated than *P. falciparum* as *P. vivax* infections recur frequently. Recurrences of *P. vivax* infections after treatment of the blood stage infection can be due to recrudescence, relapse or reinfection. The ICCs are based on baseline prevalence of *P. vivax*/*falciparum* infections and the cumulative incidence of detected *P. vivax*/*falciparum* parasitaemias at each quarterly surveys based on uPCR results.

### Estimation of *P. vivax*/*falciparum* prevalence and incidence over a 12-month period

The cumulative incidence of *P. vivax/falciparum* parasitaemias over the 12-month period was calculated based on uPCR results collected at month 0, 3, 6, 9 and 12. A participant was considered to have a recurrent *P. vivax* infection if there were two or more positive uPCR results during the 12 months follow up period. As consecutive positive uPCR tests could be due to a re-infection following a new mosquito bite or a continuous infection which is likely to be due to persistence in *P. falciparum* infections and a relapse in *P. vivax* infections. To address this uncertainty, an “episode” was defined in two ways. In the first approach each positive uPCR test was considered as a separate episode (i.e. reinfections). In the second, consecutive positive uPCR results were considered to belong to the same continuous infection (persistent or relapsing asymptomatic parasitaemia). The ICCs for the second approach are presented in the Additional file 1.

### Statistical methodology

The outcomes of interest for the ICC estimation are the prevalence and the incidence of *P. falciparum* and *P. vivax* infections. A logistic regression model is the most relevant model for prevalence while the Poison model is the most natural model when modeling incidence as the outcome of interest. The basic ICC formula presented above ($\rho = \frac{{\sigma_{b}^{2} }}{{\sigma_{b}^{2} + \sigma_{w}^{2} }}$) refers to a case where two-level hierarchical data are of interest. In practice, often several levels of clustering are available and of interest. The hierarchical structure of the data in the TME study included 4 levels: longitudinal data on infection status (level 1) collected repeatedly for each individual (level 2) who belonged to a village (level 3) which was located in a country (level 4). However, country specific ICCs were estimated because there was considerable heterogeneity in baseline *P. falciparum*/*P. vivax* prevalence between countries. In this case, the level of country is not considered. The model for estimating ICC for prevalence is reduced to 2 levels in each country because ICCs were estimate at baseline only and, therefore, each individual contributes only one observation at baseline. By contrast, for the estimation of ICC for incidence, multiple outcomes were aggregated, i.e. each individual had one observation for the outcome counts over time and the exposure time was aggregated for each individual. Two-level hierarchical models were fitted to estimate country specific ICCs for both prevalence and incidence with a village as unit of randomization.

Methodological approaches have been developed describing procedures used to compute ICC values applicable to models with multiple hierarchical levels that include logistic regression models as well as other generalized linear models such as the Poisson regression models [8, 10, 11]. The ICC values can be estimated from model equations as exact estimation methods or through use of simulations. A latent variable approach is another method for estimating ICC from logistic (logit link-scale) regression models. The link-scale is often considered to be of interest for prevalence, because the estimates of the individual outcomes are performed on the underlying latent scale [17, 18]. Nakagawa et al. provided comprehensive methods for calculating ICCs using both exact and latent variable methods for logistic and Poisson models [18]. However, Austin et al. have shown that there is no latent response formulation for Poisson models and such a model is, therefore, not included in this paper. The exact estimation method and the simulation method were utilized in the estimation of the ICC values for both incidence (Poisson model) and prevalence (logit model) of *P. falciparum* and *P. vivax*. In addition, the latent variable estimates of ICC for logit model are provided in the additional material for the estimation of ICCs for prevalence of *P. falciparum* and *P. vivax*. ICC values for the model are provided with and without the covariates sex and age because they were independently associated with the outcome [12]. The estimation of the country specific ICC is the main focus of this article. However, the overall ICC is also included for prevalence of *P*. *falciparum* and *P. vivax* using the latent variable method. For prevalence ICC uses the baseline prevalence as this is the time-point measure most often used in sample size calculations. The statistical methodology for estimating ICC from a random effects logistic model using the exact calculation, simulation-based and latent variable method is introduced. In addition the methodology for estimating ICCs from the random effect Poisson model using exact calculation and simulation-based methods is described [8].

For prevalence outcome, consider a logistic model for the outcome $Y_{ij}$, where $i$ denotes an individual and $j$ denotes a cluster then:

1 $$Y_{ij} \sim {\text{Bernoulli}}\left( {p_{ij} } \right)$$

where $p_{ij}$ is the probability of experiencing an outcome for individual $i$ in cluster $j$. And the logistic regression model is fitted as a generalized linear mixed model (GLMM) with logit link function as:

2 $${\text{logit (}}p_{ij} ) {\text{ = log}}\left( {\frac{{p_{ij} }}{{1 - p_{ij} }}} \right) = \alpha_{j} + \beta X_{ij}$$

where $X_{ij}$ refers to covariates such as age and sex measured on the individual $i$ in cluster $j$ and $\alpha_{j}$ is a cluster-specific random effect such that $\alpha_{j}$ follows a normal distribution with a mean of 0 and variance equal to $\sigma_{\alpha }^{2}$.

The exact ICC for prevalence using logistic model is calculated as follows [10]

3 $${\text{ICC}} = \frac{{\left[ {\sigma_{\alpha }^{2} p_{ij}^{2} /\left( {1 + \exp \left( {2\beta X + \sigma_{\alpha }^{2} } \right)} \right)} \right]}}{{\left[ {\sigma_{\alpha }^{2} p_{ij}^{2} /\left( {1 + \exp \left( {2\beta X + \sigma_{\alpha }^{2} } \right)} \right)} \right] + \left[ {p_{ij} \left( {1 - p_{ij} } \right)} \right]}} ,{\text{ where}}\;p_{ij} = \frac{{\exp \left( {\beta X} \right)}}{{1 + \exp \left( {\beta X} \right)}}$$

where $\sigma_{\alpha }^{2}$ (the random effect variance for the level of interest) and $\beta$ [the log (odds ratio)] are estimated from the model and $X$ refers to covariates such as age and sex.

The calculation of ICC as a postestimation estimate from software is provided using the latent variable approach for logistic model. In the logistic model, the underlying logistic error distribution has a constant variance $\frac{{\pi^{2} }}{3}$ which was used as residual variance when calculating ICC from a logistic model and then the latent ICC is given by $ICC = \frac{{\sigma_{\alpha }^{2} }}{{\sigma_{\alpha }^{2} + \frac{{\pi^{2} }}{3}}}$, where $\sigma_{\alpha }^{2}$ is the between cluster variance for the binary outcome. Goldstein et al. [11] suggested that the latent variable approach to estimate the ICC is only appropriate when the binary outcome can be an underlying continuous latent variable. However, this is the version of ICC that is readily obtained in most software including Stata. In a Poisson model, the error variance is not constant and depends on the covariates included in the model. Consider a Poisson model for the outcome $Y_{ij}$, where $i$ denotes an individual and $j$ denotes a cluster then:

4 $$Y_{ij} \sim {\text{Poisson}}\left( {\lambda_{ij} } \right)$$

And the Poisson regression model is fitted as a generalized linear mixed model (GLMM) with log link function as:

5 $${ \log }\left( {\lambda_{ij} } \right) = \alpha_{j} + \beta X_{ij}$$

where $X_{ij}$ refers to covariates such as age and sex measured on the individual $i$ in cluster $j$; $\lambda_{ij}$ is an estimate of the expected number of outcome events for individual $i$ in cluster $j$; and $\alpha_{j}$ is a cluster-specific random effect such that $\alpha_{j}$ follows a normal distribution with a mean of 0 and variance equal to $\sigma_{\alpha }^{2}$. The estimate of the ICC from exact calculation is calculated as follows [8]:

6 $${\text{ICC}} = \frac{{\left[ {\exp \left( {2\beta X + 2\sigma_{\alpha }^{2} } \right) - \exp \left( {2\beta X + \sigma_{\alpha }^{2} } \right)} \right]}}{{\left[ {\exp \left( {2\beta X + 2\sigma_{\alpha }^{2} } \right) - \exp \left( {2\beta X + \sigma_{\alpha }^{2} } \right)} \right] + \left[ {\exp \left( {\beta X + \frac{{\sigma_{\alpha }^{2} }}{2}} \right)} \right]}}$$

where $\sigma_{\alpha }^{2}$ (the random effect variance for the level of interest) and $\beta$(the log (incidence rate ratio)) are estimated from the model and $X$ refers to covariates such as age and sex. The simulation procedures are detailed in Austin et al. [8].

The simulation-based algorithm for both prevalence and incidence proceeds as follows:

1. Fit a multilevel logistic or Poisson model to an existing dataset. If the dataset is not available, one can use desired parameters estimated from previous studies to generate a dataset that mimics the original study data.
2. Simulate a large number say, *M*, cluster-level random effects ($\alpha_{m} , m = 1, \ldots ,M)$ from a mixed effect model distribution obtained from the model fitted in step 1. Typically *M *= 1000; 5000; or 10, 000 simulations but higher numbers of *M* are also possible. (5000 simulations or more may take days, weeks or months depending on the data and the fitted model.)
3. Use each of the simulated mixed effects model drawn in step 2 to compute the predicted means denoted as: ($\left( {\hat{E}\left( {Y_{m} } \right)} \right)$, and variances denoted as: $\hat{V}\left( {Y_{m} } \right)$,
4. The predicted mean is calculated as: $\hat{E}\left( {Y_{m} } \right) = \hat{p}_{m} = \frac{{\exp \left( {\hat{\beta }X + \alpha_{m} } \right)}}{{1 + \exp \left( {\hat{\beta }X + \alpha_{m} } \right)}}$ and the variance is $\hat{V}\left( {Y_{m} } \right) = \hat{p}_{m} \left( {1 - \hat{p}_{m} } \right)$ for logistic, where $\hat{p}_{m}$ is the probability of outcome predicted from the random effects model.
5. The predicted mean and variance for the Poisson model are calculated as: $\hat{E}\left( {Y_{m} } \right) = \hat{V}\left( {Y_{m} } \right) = \hat{\lambda }_{m} = \exp \left( {\hat{\beta }X + \alpha_{m} } \right)$.
6. Then compute the between cluster variance, $\hat{\sigma }_{\alpha }^{2} = V\left( {\hat{E}\left( {Y_{m} } \right)} \right)$, variance in the distribution of $\left( {\hat{E}\left( {Y_{m} } \right)} \right)$ from the mean of the $\left( {\hat{E}\left( {Y_{m} } \right)} \right)$ from the M simulations.
7. Calculate the error variance as the mean of the estimated outcome variances $E\left( {\hat{V}\left( {Y_{m} } \right)} \right) = \frac{1}{M}\sum\nolimits_{m = 1}^{M} {\hat{V}\left( {Y_{m} } \right)}$
8. Thus, the between cluster variance is $V\left( {\hat{E}\left( {Y_{m} } \right)} \right)$ while the total variance is $V\left( {\hat{E}\left( {Y_{m} } \right)} \right) + \frac{1}{\text{M}}\sum\nolimits_{m = 1}^{M} {\hat{V}\left( {Y_{m} } \right)}$.
9. The estimate of the ICC from simulation-based approach is calculated as follows [8]:
  $${\text{ICC}} = \frac{{V\left( {\hat{E}\left( {Y_{m} } \right)} \right)}}{{V\left( {\hat{E}\left( {Y_{m} } \right)} \right) + \frac{1}{\text{M}}\sum\limits_{m = 1}^{M} {\hat{V}\left( {Y_{m} } \right)} }}$$

The confidence intervals for the ICCs were calculated by using bootstrapped samples to estimate the standard error. All analyses including simulations and bootstrapping were performed in Stata 15.

## Results

The main outcomes for the TME trial were the prevalence and the incidence of *P. falciparum* infection. The overall mean uPCR prevalence of *P. falciparum* infection at baseline from the four countries was 6.2% with high heterogeneity between villages (lowest is Cambodia with 2% and highest is Laos with 11%). The incidence of *P. falciparum* parasitaemia over 12 months for intervention *vs* control arm were 28 vs 58/1000 person-years. The overall prevalence of *P. vivax* infection at baseline from the four countries was 10.3%. The incidence of *P. vivax* over 12 months for intervention *vs* control arm were 61 vs 104/1000 person-years. The data at month 12 from the control arm in Myanmar are not included in the analysis as cross-over MDA took place at month 9.

### The estimates of ICC values for prevalence of *P. falciparum* and *P. vivax*

The ICC values for the prevalence of *P. falciparum* were less than 0.10 in all countries for a model without covariates as well as the model with age and sex as covariates (Table 1). Laos had the highest ICC values for the prevalence of *P. falciparum* infection with a value of 0.08 (95% CI 0.06 to 0.11) in either model. However, these ICCs are practically similar in all the four countries.

**Table 1:** Intracluster correlation coefficient (ICC) for prevalence of *P. falciparum* infection at baseline by country, using exact calculation approach

Country	N	Intra-cluster correlation coefficient (ICC), 95% CI	
		Model without covariates	Model with covariates: sex, age
Vietnam	2301	0.003 (0.000, 0.009)	0.004 (0.000, 0.011)
Cambodia	1244	0.005 (0.000, 0.013)	0.006 (0.000, 0.014)
Myanmar	1539	0.035 (0.016, 0.054)	0.034 (0.015, 0.054)
Laos	1661	0.082 (0.056, 0.108)	0.081 (0.056, 0.107)

Similarly, as shown in Table 2, the ICC values for the prevalence of *P. vivax* infection at baseline were less than 0.10 for all countries for a model without covariates as well as the model with age and sex as covariates. Laos had the highest ICC for the prevalence of *P. vivax* infection of about 0.06 (95% CI 0.05 to 0.08). Again, these ICCs are very similar across the four countries.

**Table 2:** Intracluster correlation coefficient (ICC) for prevalence of *P. vivax* infection at baseline by country, using exact calculation approach

Country	N	Intra-cluster correlation coefficient (ICC), 95% CI	
		Model without covariates	Model with covariates: sex, age
Vietnam	2301	0.001 (0.000, 0.006)	0.005 (0.000, 0.007)
Cambodia	1244	0.020 (0.003, 0.037)	0.018 (0.002, 0.034)
Myanmar	1539	0.004 (0.000, 0.015)	0.005 (0.000, 0.016)
Laos	1661	0.061 (0.045, 0.078)	0.061 (0.045, 0.078)

### The estimates of ICC values for the incidence of *P. falciparum* and *P. vivax* parasitaemia

Using an exact calculation approach from the Poisson model for incidence of *P. falciparum*, the country specific ICC values were less than 0.02 in Vietnam, Cambodia and Myanmar for a model without covariates as well as the model with age and sex as covariates (Table 3). Laos had the highest ICC for *P. falciparum* infection 0.71 (95% CI 0.52 to 0.89). Similarly, as shown in Table 4 below the ICC values for *P. vivax* infection were very low in Vietnam, Cambodia and Myanmar i.e. ICC of less than 0.10. Laos had the highest ICC for the incidence of *P. vivax* infections of around 0.81 (95% CI 0.59 to 1.00) for a model without covariates as well as the model with age and sex as covariates.

**Table 3:** Intracluster correlation for incidence of *P. falciparum* infection by country, using exact calculation approach

Country	N	Intra-cluster correlation coefficient (ICC), 95% CI	
		Model without covariates	Model with covariates: sex, age
Vietnam	2301	0.003 (0.000, 0. 009)	0.004 (0.000, 0.011)
Cambodia	1244	0.002 (0.000, 0.267)	0.003 (0.000, 0.220)
Myanmar	1539	0.011 (0.000, 0.069)	0.013 (0.000, 0.079)
Laos	1661	0.707 (0.523, 0.892)	0.701 (0.514, 0.889)

**Table 4:** Intracluster correlation for incidence of *P. vivax* infection by country, using exact calculation approach

Country	N	Intra-cluster correlation coefficient (ICC), 95% CI	
		Model without covariates	Model with covariates: sex, age
Vietnam	2301	0.008 (0.000, 0.018)	0.009 (0.000, 0.019)
Cambodia	1244	0.075 (0.002, 0.149)	0.075 (0.003, 0.147)
Myanmar	1539	0.003 (0.000, 0.010)	0.003 (0.000, 0.010)
Laos	1661	0.806 (0.592, 1.000)	0.804 (0.587, 1.000)

### Simulation and latent variable vs exact calculation methods for estimation of ICCs for prevalence/incidence of *P. falciparum* and *P. vivax*

**Fig. 1:**
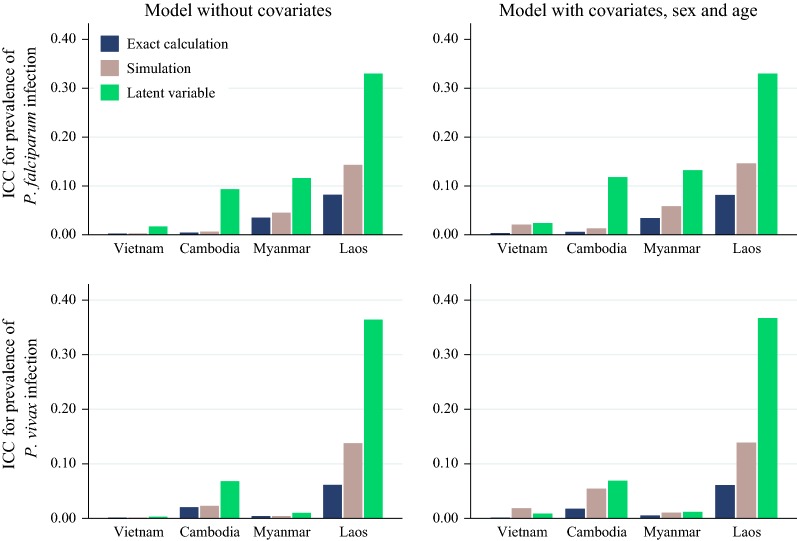
Intracluster correlation coefficient (ICC) for prevalence of *P. falciparum* and *P. vivax* infection by country and by estimation methods

The actual ICC values from simulation and latent variable approaches are presented in the Additional file 1: Tables S1–S6. Simulations gave consistently higher ICC values than the corresponding exact calculation method for prevalence (Fig. 1). However, exact calculation gave lower ICC than latent variable approach for prevalence. In fact, the latent variable gave the highest ICCs compared to both the exact and the simulation methods for prevalence. The same trend was observed for estimation of ICCs for incidence with simulations giving consistently higher ICC values than the corresponding the exact calculation method (Additional file 2: Figures S1).

The overall estimated ICC from the latent variable approach for the prevalence are 0.26 (95% CI 0.13 to 0.45) and 0.21 (95% CI 0.10 to 0.38) for *P. falciparum* and *P. vivax* (in the 16 villages), respectively.

### Sensitivity analysis results

As shown in the Additional file 1: Tables S7–S10, the estimates of ICC are generally similar to corresponding scenarios under definitions of incidence.

### Illustration of the impact of ICCs on cluster sizes and implications for design of Malaria pre-elimination studies in the Greater Mekong Subregion

In order to illustrate the number of clusters/villages (sample sizes) that would be needed to design new malaria pre-elimination trial, the overall ICC was estimated for the four countries using latent method for the comparison of prevalence of *P. falciparum* and *P. vivax* between the control and the intervention. The overall estimated ICCs from the latent variable approach are 0.26 (95% CI 0.13 to 0.45) and 0.21 (95% CI 0.10 to 0.38) for *P. falciparum* and *P. vivax* (in the 16 villages) respectively. Baseline prevalence observed in the TME trial for each country was used as the control prevalence. In line with the parent trial, the aim was to detect at least a 95% decline in prevalence of *P. falciparum* following administration of MDA plus a single low dose primaquine. The mean village size was set at 500 participants in line with the observed number of participants per village in the TME trial. Table 5 summarizes the expected number of villages per arm required in each country for stand-alone studies. For the design of a multicentre cluster randomized trial for the Greater Mekong Subregion the overall sample size is provided based on the average prevalence across the four countries. Separate cluster randomized trials, need to recruit 134 villages per arm for Cambodia due to the low prevalence.

**Table 5:** Required number of villages per study arm using the observed ICCs based on the latent variable approach from TME trial to detect a 95% fall in prevalence of *P. falciparum* and 99% fall in prevalence of *P. vivax* from a baseline prevalence from TME trial with 80% power, 0.05 probability of Type I error and cluster size of 500 participants

Country	Prevalence (control) (%)	Prevalence (intervention) (%)	Number of villages/arm
*P. falciparum*			
Vietnam	4.0	0.2	59
Cambodia	1.8	0.1	134
Laos	10.9	0.5	21
Myanmar	8.0	0.4	29
Overall	6.2	0.3	37
*P. vivax*			
Vietnam	6.8	0.1	25
Cambodia	9.6	0.1	17
Laos	8.3	0.1	20
SMRU	18.1	0.2	9
Overall	10.3	0.1	16

**Fig. 2:**
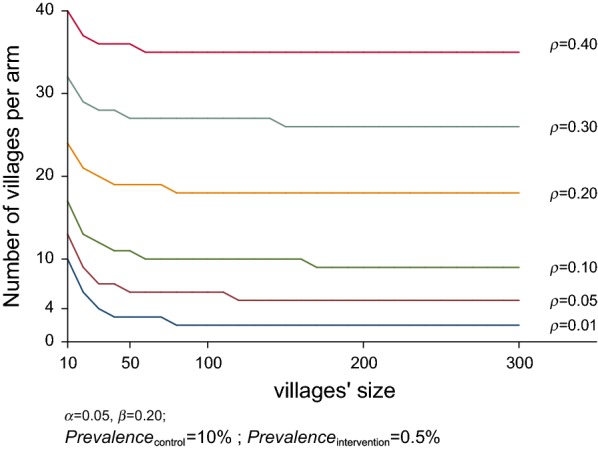
Required number of villages for varying village sizes for the different ICCs (rho) assuming to detect a 95% fall in prevalence of *P. falciparum* from a 10% initial prevalence (control groups) with 80% power and 0.05 probability of Type I error

The required number of clusters varies with varying villages sizes. As expected, the number of clusters increase with increasing ICC for a constant number of individuals per village. Figure 2 also shows that for ICCs between 0 and 0.4, with the given effect size, increasing the number of resident per village (village size) above 50 does not result in an increased benefit in terms of the number of clusters (villages) required to have sufficient statistical power.

## Discussion

The ICCs were estimated for the prevalence as well as the incidence of *P. falciparum* and *P. vivax* infections in Southeast Asia. The baseline ICCs for estimation of prevalence of *P. vivax* were generally very low in all countries using the exact calculation method. Use of the latent variable approach resulted in very high ICC for Laos but still very low for other countries. The very high ICCs observed in Laos highlights the danger of studying few clusters as there can be very big differences between cluster variances by chance alone. It is, therefore, advisable to survey a sufficiently large number of villages in a pilot screening phase in order to understand the heterogeneity to be expected during the randomization. For example, the mean baseline *P. falciparum* prevalence in the villages randomized to intervention was 4.8% while that of the control villages was 17.5% in Laos. The high prevalence in Laos mean was driven by a single village where the prevalence of *P. falciparum* was 28.8% while in the other control village it was 1.2%. Similarly, the mean baseline *P. vivax* prevalence in the villages randomized to intervention was very low, i.e. 2.3% while that of the control villages was 14.7% in Laos. The low ICC for the prevalence of *P. falciparum* in all countries from the exact calculation method is consistent with the sample size that was used in the TME study for that site. Based on limited resources only four villages were studied in each of the four participating countries. There remains a lack of generalizability when only few clusters are studied. ICCs for the estimation of *P. vivax* and *P. falciparum* incidence were in general low in all countries except for Laos. The ICCs from simulations were higher than those from the corresponding exact calculation methods. Since our ICCs have a wide range, they may be used under a wide range of circumstances. In case of uncertainty the use of high ICCs obtained for Laos and from the latent variable approach for prevalence are best suited to avoid insufficiently powered trials.

The size of the ICCs depends on the method of estimation that is used to calculate the ICCs. In general, the latent variable method tends to lead to higher ICC estimates compared to exact and simulation methods. This has implications for the design effect. Estimates of ICCs from the exact methods will lead to lower design effects than simulations or latent variable approaches. Hence smaller sample size estimates will be obtained using the ICCs from the exact calculation or simulation methods than the latent variable approach. Researchers need to consider whether the assumptions underlying the distribution of the outcome are reasonable with reference to the estimation method. Where possible researchers should use the more conservative latent variable approach for resulting in higher prevalence ICCs a higher design effect and hence higher sample size estimates relative to other methods. A simulation-based method may be used to obtain conservative estimates of ICC for the estimation of incidence instead of the exact method. Where researchers are confident of the assumptions underlying the distribution of the outcome, the exact, latent variable or simulation-based method should be applied as appropriate in line with the assumptions.

Where appropriate data are available, the latent variable approach for logistic models and exact method for Poisson models should be used as it is more transparent for the reader. Simulations should only be used when data is limited. It should be noted that the influence of ICCs on sample sizes also depends on the cluster randomized trial design that is planned. A conventional parallel cluster randomized trial design will require a smaller sample size with decreasing ICCs while the stepped wedge design operates in an exact opposite way. For a stepped wedge study design, the sample size first slightly increases with increasing ICC up to about ICC of 0.05, and then starts decreasing. Thus, the highest sample size is obtain with an ICC of 0.05 [19]. It is important to have an understanding of the ICCs in order to design studies appropriately as rules of thumb may not apply to all types of cluster randomized trials and underpowered studies run the risk of being futile or provide spurious negative results. This study focuses on detection of *Plasmodium* infections detected by uPCR and not on clinical malaria episodes. In the absence of clinical data, it is difficult to assess whether this limitation has an effect on ICCs which use clinical outcomes as endpoint.

## Conclusion

This study provides a range of ICC values that can aid in calculation of sample sizes for cluster randomized trials relying on outcomes of *P. vivax* or *P. falciparum*. Researchers should use the ICCs that are based on exact/latent method when enough data is available. Where researchers plan multicountry studies, getting may be best to base sample size estimates on the mean of these ICCs. Similarly, for countries that are close to these regions but were not part of the study, they can use the mean estimates. Those planning to use stepped-wedge design should use the lowest values, especially an ICC value of 0.05 while those planning parallel cluster randomized trials may wish to use the highest values of ICCs so as to avoid underpowered trials. Use of mean values may be appropriate in situations where extreme values result in unreasonably high or low numbers of clusters. As malaria transmission is changing, researchers should report ICCs when publishing their work to aid the design of future trials.

## Supplementary information

**Additional file 1.** Additional tables.

**Additional file 2: Fig. S1.** Intracluster correlation for incidence of *P. falciparum* and *P. vivax* infection using exact calculation and simulation-based approach from model without covariates.

## Data Availability

The data is available upon request to the Mahidol Oxford Tropical Medicine Research Unit Data Access Committee (http://www.tropmedres.ac/data-sharing) complying with the data access policy (http://www.tropmedres.ac/_asset/file/data-sharing-policy-v1-0.pdf).

## Abbreviations

CRTs: cluster randomized trials
DEff: design effect
ICC: intracluster correlation coefficient
MDA: mass drug administration
TME: targeted malaria elimination study
uPCR: ultrasensitive quantitative PCR
VIF: variance inflation factor
