# Supplementary material for: Intracluster correlation coefficients in the Greater Mekong Subregion for sample size calculations of cluster randomized malaria trials
Source: Malar J. 2019 Dec 18;18:428. doi: 10.1186/s12936-019-3062-x (PMC6921387; doi:10.1186/s12936-019-3062-x)
Supplement: Supplementary file 1 — Additional file 1. Additional tables. [file 12936_2019_3062_MOESM1_ESM.docx]

**Table S1** Intracluster correlation for incidence of *P. falciparum* infection by country, using simulation-based approach

| Country | N | Intra-cluster correlation coefficient (ICC) , 95% CI | |
| --- | --- | --- | --- |
|  |  | Model without covariates | Model with covariates: sex, age |
| Vietnam | 2,301 | 0.004 (0.026, 0.045) | 0.072 (0.050, 0.093) |
| Cambodia | 1,244 | 0.007 (0.000, 0.280) | 0.014 (0.000, 0.272) |
| Myanmar | 1,539 | 0.021 (0.000, 0.099) | 0.042 (0.000, 0.167) |
| Laos | 1,661 | 0.745 (0.575, 0.915) | 0.742 (0.573, 0.911) |

**Table S2** Intracluster correlation for incidence of *P. vivax* infection by country, using simulation-based approach

| Country | N | Intra-cluster correlation coefficient (ICC) , 95% CI | |
| --- | --- | --- | --- |
|  |  | Model without covariates | Model with covariates: sex, age |
| Vietnam | 2,301 | 0.056 (0.044, 0.069) | 0.114 (0.087, 0.140) |
| Cambodia | 1,244 | 0.133 (0.027, 0.239) | 0.187 (0.063, 0.310) |
| Myanmar | 1,539 | 0.083 (0.072, 0.093) | 0.107 (0.088, 0.126) |
| Laos | 1,661 | 0.828 (0.623, 1.000) | 0.824 (0.633, 1.000) |

**Table S3** Intracluster correlation coefficient (ICC) for prevalence of *P. falciparum* infection at baseline by country, using simulation-based approach

| Country | N | Intra-cluster correlation coefficient (ICC) , 95% CI | |
| --- | --- | --- | --- |
|  |  | Model without covariates | Model with covariates: sex, age |
| Vietnam | 2,301 | 0.003 (0.000, 0. 011) | 0.021 (0.003, 0.038) |
| Cambodia | 1,244 | 0.006 (0.000, 0.054) | 0.013 (0.000, 0.074) |
| Myanmar | 1,539 | 0.045 (0.013, 0.077) | 0.058 (0.065, 0.228) |
| Laos | 1,661 | 0.143 (0.061, 0.225) | 0.146 (0.023, 0.094) |

**Table S4** Intracluster correlation coefficient (ICC) for prevalence of *P. vivax* infection at baseline by country, using simulation-based approach

| Country | N | Intra-cluster correlation coefficient (ICC) , 95% CI | |
| --- | --- | --- | --- |
|  |  | Model without covariates | Model with covariates: sex, age |
| Vietnam | 2,301 | 0.001 (0.000, 0.007) | 0.019 (0.004, 0.033) |
| Cambodia | 1,244 | 0.023 (0.000, 0.046) | 0.054 (0.026, 0.083) |
| Myanmar | 1,539 | 0.004 (0.000, 0.014) | 0.011 (0.000, 0.024) |
| Laos | 1,661 | 0.138 (0.056, 0.219) | 0.139 (0.058, 0.220) |

**Table S5** Intracluster correlation coefficient (ICC) for prevalence of *P. falciparum* infection at baseline by country, using the latent variable approach

| Country | N | Intra-cluster correlation coefficient (ICC), 95% CI | |
| --- | --- | --- | --- |
|  |  | Model without covariates | Model with covariates: sex, age |
| Vietnam | 2,301 | 0.017 (0.001, 0.180) | 0.024 (0.003, 0.186) |
| Cambodia | 1,244 | 0.093 (0.005, 0.692) | 0.118 (0.008, 0.699) |
| Myanmar | 1,539 | 0.116 (0.028, 0.374) | 0.132 (0.032, 0.408) |
| Laos | 1,661 | 0.330 (0.102, 0.682) | 0.330 (0.102, 0.681) |

**Table S6** Intracluster correlation coefficient (ICC) for prevalence of *P. vivax* infection at baseline by country, using the latent variable approach

| Country | N | Intra-cluster correlation coefficient (ICC) , 95% CI | |
| --- | --- | --- | --- |
|  |  | Model without covariates | Model with covariates: sex, age |
| Vietnam | 2,301 | 0.003 (0.001, 0.381) | 0.006 (0.001, 0.154) |
| Cambodia | 1,244 | 0.068 (0.014, 0.272) | 0.061 (0.012, 0.258) |
| Myanmar | 1,539 | 0.010 (0.001, 0.078) | 0.010 (0.001, 0.084) |
| Laos | 1,661 | 0.364 (0.117, 0.712) | 0.363 (0.116, 0.711) |

**Sensitivity analysis**

**Table S7** Intracluster correlation for incidence of *P. falciparum* infection by country, using exact calculation approach

| Country | N | Intra-cluster correlation coefficient (ICC) , 95% CI | |
| --- | --- | --- | --- |
|  |  | Model without covariates | Model with covariates: sex, age |
| Vietnam | 2,301 | 0.001 (0.000, 0. 006) | 0.002 (0.000, 0.007) |
| Cambodia | 1,244 | 0.003 (0.000, 0.268) | 0.004 (0.000, 0.269) |
| Myanmar | 1,539 | 0.005 (0.000, 0.063) | 0.006 (0.000, 0.072) |
| Laos | 1,661 | 0.494 (0.310, 0.678) | 0.492 (0.308, 0.677) |

**Table S8** Intracluster correlation for incidence of *P. falciparum* infection by country, using simulation-based approach

| Country | N | Intra-cluster correlation coefficient (ICC) , 95% CI | |
| --- | --- | --- | --- |
|  |  | Model without covariates | Model with covariates: sex, age |
| Vietnam | 2,301 | 0.030 (0.023, 0.036) | 0.052 (0.039, 0.066) |
| Cambodia | 1,244 | 0.007 (0.000, 0.182) | 0.019 (0.000, 0.224) |
| Myanmar | 1,539 | 0.012 (0.000, 0.056) | 0.030 (0.000, 0.100) |
| Laos | 1,661 | 0.536 (0.299, 0.773) | 0.533 (0.297, 0.769) |

**Table S9** Intracluster correlation for incidence of *P. vivax* infection by country, using exact calculation approach

| Country | N | Intra-cluster correlation coefficient (ICC) , 95% CI | |
| --- | --- | --- | --- |
|  |  | Model without covariates | Model with covariates: sex, age |
| Vietnam | 2,301 | 0.004 (0.000, 0.010) | 0.004 (0.000, 0.010) |
| Cambodia | 1,244 | 0.037 (0.000, 0.110) | 0.037 (0.000, 0.109) |
| Myanmar | 1,539 | 0.003 (0.000, 0.010) | 0.004 (0.000, 0.011) |
| Laos | 1,661 | 0.642 (0.429, 0.855) | 0.640 (0.424, 0.856) |

**Table S10** Intracluster correlation for incidence of *P. vivax* infection by country, using simulation-based approach

| Country | N | Intra-cluster correlation coefficient (ICC) , 95% CI | |
| --- | --- | --- | --- |
|  |  | Model without covariates | Model with covariates: sex, age |
| Vietnam | 2,301 | 0.038 (0.030, 0.046) | 0.085 (0.066, 0.104) |
| Cambodia | 1,244 | 0.074 (0.018, 0.149) | 0.110 (0.043, 0.177) |
| Myanmar | 1,539 | 0.078 (0.070, 0.086) | 0.078 (0.063, 0.092) |
| Laos | 1,661 | 0.679 (0.372, 0.984) | 0.678 (0.374, 0.982) |
