# Supplementary figures and images for: Intracluster correlation coefficients in the Greater Mekong Subregion for sample size calculations of cluster randomized malaria trials
Source: Malar J. 2019 Dec 18;18:428. doi: 10.1186/s12936-019-3062-x (PMC6921387; doi:10.1186/s12936-019-3062-x)

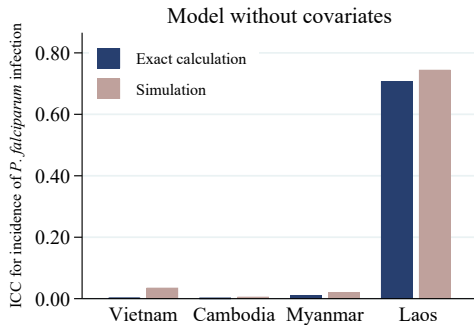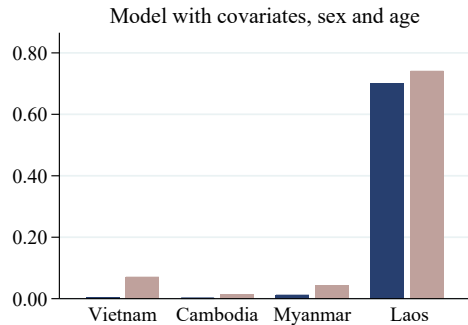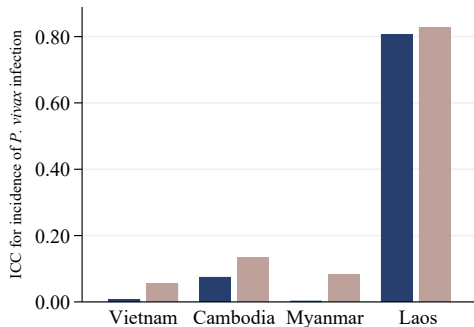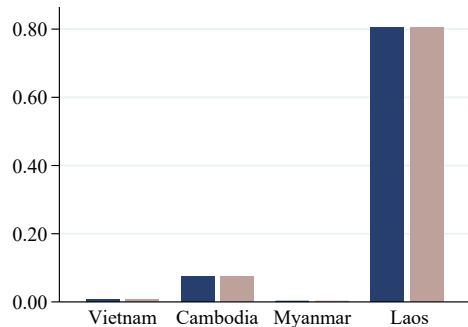

Supplement: Supplementary file 2 — Additional file 2: Fig. S1. Intracluster correlation for incidence of P. falciparum and P. vivax infection using exact calculation and simulation-based approach from model without covariates. [file 12936_2019_3062_MOESM2_ESM.pdf]
